# Enhancing Hair Regrowth in Pediatric Morphea en Coup de Sabre by Fractional CO_2_ Laser‐Assisted Exosome Delivery

**DOI:** 10.1111/jocd.70270

**Published:** 2025-06-04

**Authors:** Rhea Ahuja, Patrick Po‐Han Huang

**Affiliations:** ^1^ Dermatology and Aesthetics Kaohsiung Taiwan

**Keywords:** alopecia, Asian patients, cosmetic dermatological surgery, hair follicles

1

Morphea en coup de sabre (MCS) is a rare subtype of linear localized scleroderma that affects young children, presenting as a longitudinal sclerotic plaque on the scalp and forehead, often with associated hair loss [1]. Without timely intervention, sclerosis may lead to irreversible alopecia and skin atrophy. While medical therapies have shown limited success in reversing scarring alopecia, autologous fat grafting has demonstrated potential [2]. Emerging regenerative approaches, such as cell‐free therapies, may further expand treatment options.

A 6‐year‐old girl with MCS had been treated for a year with topical steroids, tacrolimus, calcipotriol, and latanoprost eye drops for hair regrowth. However, new plaques developed on the trunk and buttocks, leading to the initiation of systemic methotrexate (3.75 mg/week) and prednisolone (2.5–5 mg/day). Despite over a year of systemic therapy, hair regrowth was minimal, and sclerosis persisted in alopecic scalp areas. With limited response to conventional treatment, the family consented to a novel regenerative intervention: CO_2_ laser‐assisted exosome delivery.

Prior to the procedure, all alopecic areas were documented using dermoscopy (Heine IC1 Dermatoscope with iPod 6) and digital photography (Nikon D3200, Nikon Corporation, Tokyo, Japan). Topical anesthesia was achieved with a eutectic mixture of lidocaine 2.5% and prilocaine 2.5% under occlusion for 30 min.

A solution of rose (Rosa Damascena) stem cell‐derived exosomes (SCE) was prepared by combining 2 mL normal saline with one vial of ASCE+ Derma Signal SRLV‐S (ExoCoBio Inc., Seoul, Republic of Korea). After disinfection with 70% alcohol, fractional CO_2_ laser treatment (eCO_2_, Lutronic Inc., Goyang, Republic of Korea) was performed using a 10 600‐nm wavelength, 120 μm spot size, 30 mJ energy, and 100 density/cm^2^. Immediately post‐laser, the SCE solution was applied topically and occluded for 10 min to enhance transdermal delivery.

At the 14‐day follow‐up, increased follicular prominence was noted at the periphery of hairless patches. By the fourth week, follicular papules were evident, especially in the vertex and temporal regions. New grayish‐black hair growth was visible by week six (Figure 1), with mild–moderate overall improvement and thickening of pre‐existing thin hairs by week 10. Areas with dermoscopic signs of epidermal atrophy showed minimal regrowth.

**FIGURE 1:**
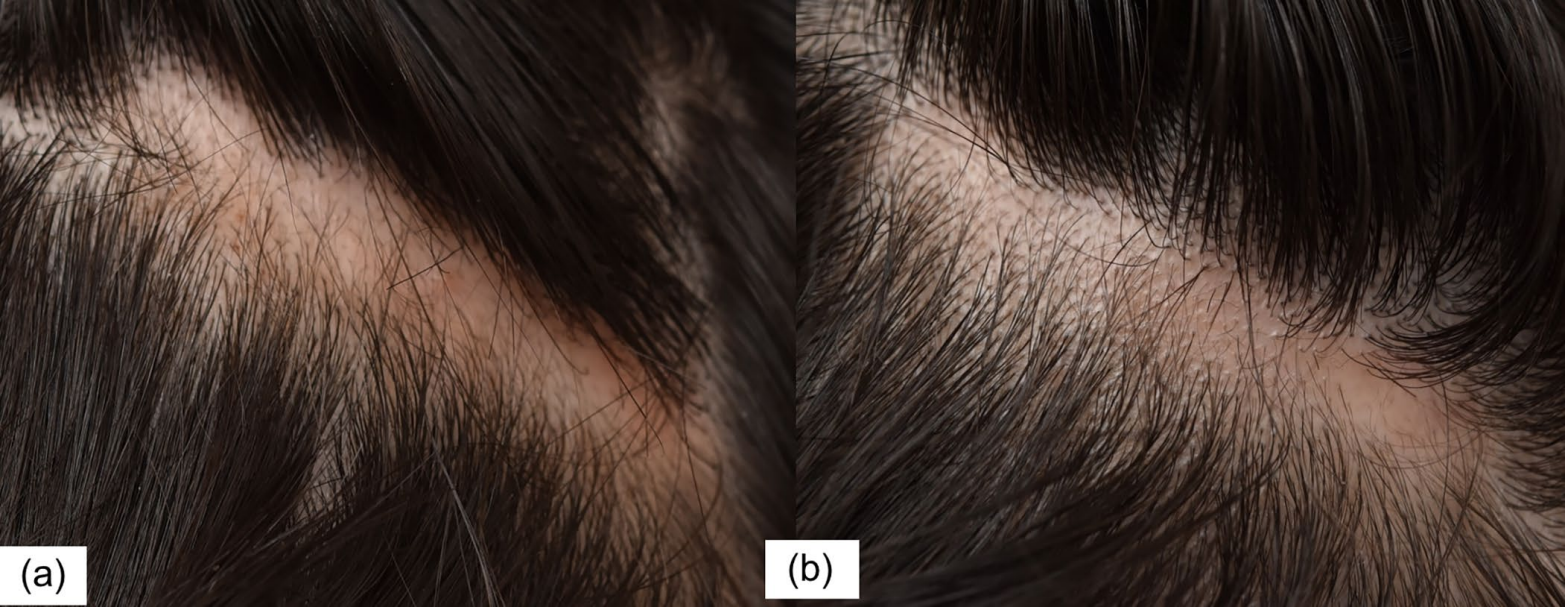
Compared to 2 weeks (a), new hair growth evident at 6‐weeks (b).

Dermoscopy of representative areas, repeated every 2 weeks, showed initial perifollicular and diffuse erythema with dilated vessels and hypopigmented spots. Post‐treatment, redness gradually diminished, with significant reduction observed by weeks 6–10. Hair regrowth ranged from mild to moderate (Figure 2) across treated sites, with variable response based on baseline dermoscopic features.

**FIGURE 2:**
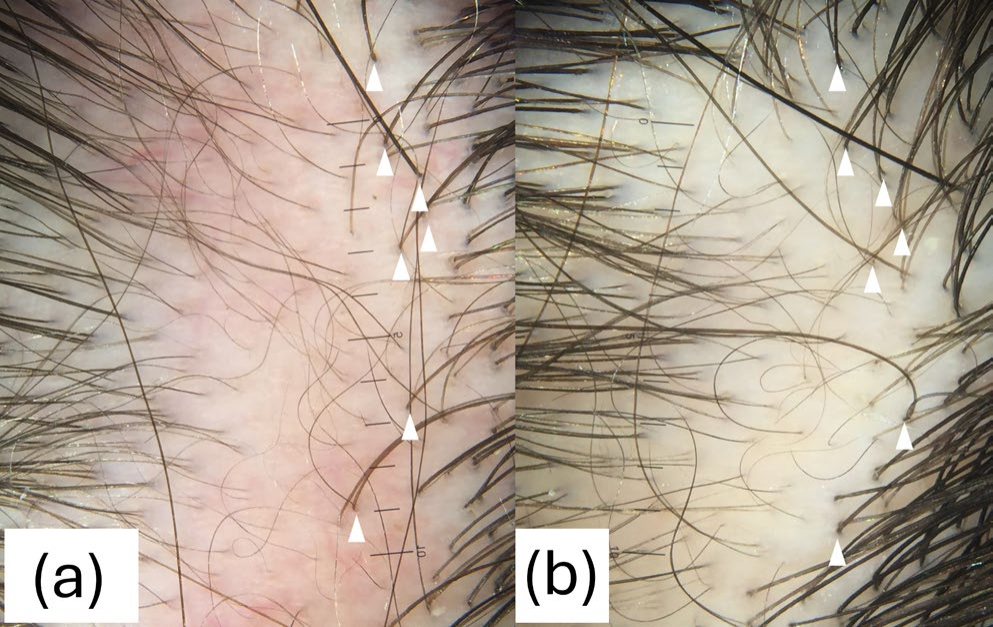
Dermoscopic view of the left parietal area near the vertex, before (a) and after 10‐weeks follow‐up (b), showing decreased redness.

Stem cell‐based approaches for alopecia are still emerging, with early studies indicating potential safety and efficacy. Among them, human‐induced pluripotent stem cells (hiPSCs) have been explored for scarring alopecia, where hair follicles are permanently lost [3]. Exosomes—vesicles rich in cytokines, growth factors, and microRNAs—may similarly modulate the hair follicle cycle and surrounding microenvironment [4].

Despite growing interest, clinical evidence for exosome therapy in alopecia is scarce. Only one study has reported its use in male pattern hair loss [5], with no documented cases in scarring alopecia, positioning this report as a possible first. Various delivery methods, such as microneedling and fractional lasers, have been employed to enhance uptake. In this case, fractional CO_2_ laser at low fluence and density was used to optimize exosome absorption while minimizing side effects. Further, the fractional laser may also stimulate the anagen growth phase via Wnt signaling and triggering follicular genesis from non‐follicular stem cells via wound healing pathways [6].

Despite promising potential, exosome therapies face regulatory challenges. The FDA has cautioned against unapproved exosome products, and no cell‐free treatment currently holds formal approval in most countries [7]. Nonetheless, early clinical studies—for androgenetic alopecia [5] and acne scars [8]—have reported favorable safety profiles without serious adverse events.

This case is limited by its single‐subject design, absence of inter‐observer validation, and uncertainty around optimal dosing. We acknowledge that outcomes may improve with additional treatment sessions, and this warrants further study. The clinical utility of exosome therapy remains under debate, especially in scarring alopecia where follicular regeneration is uncertain. However, emerging first‐in‐human reports suggest that topical exosome application—especially when combined with laser‐assisted delivery—may offer a safe, non‐invasive option in refractory skin conditions. The observed regrowth warrants further validation in larger studies.

## Author Contributions

R.A. conceptualized and designed the study, drafted the initial manuscript, and revised the manuscript. P.P.‐H.H. conceptualized and designed the study, collected data, drafted the initial manuscript, and critically reviewed and revised the manuscript.

## Consent

The parents gave written consent for the publication of the case details and photos.

## Conflicts of Interest

The authors declare no conflicts of interest.

## Data Availability

Data sharing is not applicable to this article as no new data were created or analyzed in this study.

## References

[1] H. Zaaroura , E. Pope , R. M. Laxer , and C. Sibbald , “Reversible Alopecia in En Coup de Sabre Morphea,” Pediatric Dermatology 38, no. 6 (2021): 1532–1534. 34647362 10.1111/pde.14827

[2] M. El Omari , M. Debbarh , M. A. Lakhdari , Z. Basri , and R. Ait Benhamou , “Adipose Tissue Grafting for the Treatment of Morphea En Coup De Sabre: A Simple Filler or an Emerging Cellular Therapy?,” Cureus 14, no. 10 (2022): e30358. 36407158 10.7759/cureus.30358 PMC9665929

[3] M. Ohyama , “Use of Human Intra‐Tissue Stem/Progenitor Cells and Induced Pluripotent Stem Cells for Hair Follicle Regeneration,” Inflammation and Regeneration 39 (2019): 4. 30834027 10.1186/s41232-019-0093-1 PMC6388497

[4] M. Cheng , C. Ma , H. D. Chen , Y. Wu , and X. G. Xu , “The Roles of Exosomes in Regulating Hair Follicle Growth,” Clinical, Cosmetic and Investigational Dermatology 17 (2024): 1603–1612. 38984321 10.2147/CCID.S465963 PMC11232880

[5] B. Park , H. Choi , G. Huh , and W. Kim , “Effects of Exosome From Adipose‐Derived Stem Cell on Hair Loss: A Retrospective Analysis of 39 Patients,” Journal of Cosmetic Dermatology 21, no. 5 (2022): 2282–2284. 35157363 10.1111/jocd.14846

[6] R. El‐Husseiny , S. Elframawy , and M. Abdallah , “Comparative Study Between Fractional Carbon Dioxide Laser vs Intralesional Steroid Injection in Treatment of Alopecia Areata,” Dermatologic Therapy 33 (2020): e13742. 32478930 10.1111/dth.13742

[7] U.S. Food and Drug Administration , “Public Safety Notification on Exosome Products,” 2019, https://www.fda.gov/vaccines‐blood‐biologics/safety‐availability‐biologics/public‐safety‐notification‐exosome‐products.

[8] H. Kwon , S. Yang , J. Lee , et al., “Combination Treatment With Human Adipose Tissue Stem Cell‐Derived Exosomes and Fractional CO2 Laser for Acne Scars: A 12‐Week Prospective, Double‐Blind, Randomized, Split‐Face Study,” Acta Dermato‐Venereologica 100, no. 18 (2020): adv00310, 10.2340/00015555-3666. 33073298 PMC9309822

